# Sleep Characteristics and Mood of Professional Esports Athletes: A Multi-National Study

**DOI:** 10.3390/ijerph18020664

**Published:** 2021-01-14

**Authors:** Sangha Lee, Daniel Bonnar, Brandy Roane, Michael Gradisar, Ian C. Dunican, Michele Lastella, Gemma Maisey, Sooyeon Suh

**Affiliations:** 1 Department of Psychology, Sungshin Women’s University, Seoul 02844, Korea; xrpsychology@gmail.com; 2 College of Education, Psychology and Social Work, Flinders University, Adelaide, SA 5042, Australia; daniel.bonnar@flinders.edu.au (D.B.); michael.gradisar@flinders.edu.au (M.G.); 3 UNT Health Science Centre, Department of Physiology and Anatomy, University of North Texas, Fort Worth, TX 76107, USA; brandy.roane@unthsc.edu; 4 Centre for Sleep Science, School of Human Sciences, The University of Western Australia, Perth, WA 6009, Australia; iandunican@sleep4performance.com.au; 5 Appleton Institute for Behavioral Science, Central Queensland University, Adelaide, SA 5034, Australia; m.lastella@cqu.edu.au; 6 School of Medical and Health Sciences, Edith Cowan University, Perth, WA 6027, Australia; gemma.maisey@meliusconsulting.com.au

**Keywords:** esports, sleep, mood, performance, eveningness, depression

## Abstract

Esports is becoming increasingly professionalized, yet research on performance management is remarkably lacking. The present study aimed to investigate the sleep and mood of professional esports athletes. Participants were 17 professional esports athletes from South Korea (*N* = 8), Australia (*N* = 4), and the United States (*N* = 5) who played first person shooter games (mean age 20 ± 3.5 years, 100% male). All participants wore a wrist-activity monitor for 7–14 days and completed subjective sleep and mood questionnaires. Participants had a median total sleep time of 6.8 h and a sleep efficiency of 86.4% per night. All participants had significantly delayed sleep patterns (median sleep onset 3:43 a.m. and wake time 11:24 a.m.). Participants had a median sleep onset latency of 20.4 min and prolonged wake after sleep onset of 47.9 min. Korean players had significantly higher depression scores compared to the other groups (*p* < 0.01) and trained longer per day than the Australian or United States teams (13.4 vs. 4.8 vs. 6.1 h, respectively). Depression scores were strongly correlated with number of awakenings, wake after sleep onset, and daily training time (*p* < 0.05). As the first pilot sleep study in the esports field, this study indicates that esports athletes show delayed sleep patterns and have prolonged wake after sleep onset. These sleep patterns may be associated with mood (depression) and training time. Sleep interventions designed specifically for esports athletes appear warranted.

## 1. Introduction

Esports (electronic sports) is a form of organized video game competition that has become a global phenomenon over the last decade [1]. Recent estimates indicate that there were 443 million viewers and 885 major events in 2019 [2], with prize money in some major tournaments exceeding Wimbledon and the US Masters [3]. As the esports industry has grown and become more competitive, esports athletes have been increasingly required to try and gain performance advantages over opponents [4]. However, a consequence of esports’ rapid growth is that performance-based research has not kept pace with its sharp rise in popularity. Thus, limited data exists on the factors that influence performance in esports, restricting the ability of esports athletes to make evidence-based decisions about performance management.

Due to similarities between traditional athletes and esports athletes (e.g., regular training/competition schedules and travel), the sports science literature offers some useful insights into the importance of sleep for health and performance in competitive settings. The current body of evidence suggests that optimal sleep is important for several elements (but not all) of physical performance (e.g., anaerobic power), many aspects of cognitive performance (e.g., attention) [5], and recovery (e.g., growth and repair of cells) [6]. Yet, despite the need for sleep, esports athletes and traditional athletes are both exposed to unique conditions (e.g., congested training/competition schedules) that may compromise their ability to achieve optimal sleep, potentially placing them at risk of sleep restriction and the associated consequences [1].

In contrast to many traditional sports, esports is a predominantly cognitive-based activity [7], and sleep is considered critical for optimal cognitive performance [8]. In a landmark study, Van Dongen et al. [9] investigated the cumulative effect of chronic sleep restriction on cognitive performance. They found that relative to a group who slept 8 h per day, participants who obtained 4 or 6 h of sleep per day over a 2-week period performed progressively worse on a test of reaction time and attentional lapses. In addition, recent meta-analytic evidence demonstrated that sleep restriction reliably worsens executive functioning, working memory, and sustained attention [10]. Taken together, the broad-spectrum impact of sleep restriction on cognitive performance may have important implications for performance in esports.

Parallel to the link between sleep and cognitive performance, there is a well-known relationship between disturbed sleep and affective states (e.g., [11,12,13]). For example, Dinges et al. [13] observed that participants who slept 5 h per night over a 7-day period had elevated subscale scores for fatigue, confusion, tension, and total mood disturbance on the Profile of Mood States. Accumulating evidence suggests that the restricted sleep of adolescents, as well as prolonged wakefulness in bed, is predictive of depressive symptoms and anxiety [14], a particularly pertinent finding given the young age of many esports athletes [15]. These findings are important given the proposed effect of mood on athletic performance [16] and reports of high burn out rates in esports [17].

Preliminary evidence from two recent studies [18,19] suggests that esports athletes from the United States (US) (*N* = 9) and Germany (*N* = 14) obtain the recommended 7–9 hours’ sleep per night for adults (8–10 h for teens) and have good sleep quality. However, because sleep was not the primary focus of either study, only basic single-item retrospective questions were used to measure these outcomes, and while easy to utilize, they are prone to inaccuracy [20]. Furthermore, no other data about participants’ sleep behavior (e.g., sleep timing) were collected. Notably, these findings of adequate sleep contrast with evidence demonstrating the opposite, that is, circadian timing delaying and total sleep time (TST) shortening across adolescence and into early adulthood, with many young people obtaining insufficient TST, particularly males [21]. These findings differ between regions, with individuals from Asia reporting the shortest TST and more delayed sleep patterns than their European, North American, or Oceania peers. Hence, it is possible that there may also be regional differences in the sleep behavior of esports athletes, especially from Asian countries where esports are popular.

Considering evidence in the literature showing the negative impact of sub-optimal sleep on cognitive performance and mood, it follows that sleep could be an important determinant of performance in esports. However, there are no published objective data of the sleep behavior of professional esports athletes. To address this lack of empirical evidence, two primary aims were proposed. The first was to investigate sleep and wake behaviors of esports athletes (e.g., training times and caffeine use) in countries within several global regions (i.e., Oceania, Asia, and North America) that also map onto established esports regions. The second was to examine how the sleep behavior of esports athletes in these regions (i.e., United States, Australia, and South Korea) relates to affect, specifically, measures of anxiety and depression. As the first exploratory study to specifically investigate sleep and mood, our findings will provide much-needed insight into a potentially vital aspect of performance management in arguably the fastest growing industry in the world—esports.

## 2. Materials and Methods

This was a cross-sectional, observational study and complied with the STROBE (The Strengthening the Reporting of Observational Studies in Epidemiology) guidelines for reporting of observational studies.

### 2.1. Participants

The 17 participants were South Korean (*N* = 8), Australian (*N* = 4), and United States (US) (*N* = 5) elite esports athletes competing in First Person Shooter games [15] within a professional league. Ethics approval was obtained in all three countries from relevant university ethics boards (Flinders University Social and Behavioral Research Ethics Committee, Sungshin Women’s University Review Board, and the University of North Texas’ Institutional Review Board). All participants provided written informed consent.

#### Inclusion/Exclusion Criteria

Participants were eligible for inclusion if they competed as part of a professional team within an official esports league. Participants were excluded if they did not complete the entire protocol, withdrew consent, or did not regularly wear the wrist-activity monitor resulting in unreliable sleep data.

### 2.2. Measures

#### 2.2.1. Demographic/General Information

A self-report questionnaire measured demographic and general information, including, self-reported anthropometric data (i.e., height and weight), esports background (e.g., number of years playing professionally), sleep history (e.g., sleep disturbance prior to competition), and level of exposure to risk factors known to affect the sleep of athletes (e.g., caffeine use and training time).

#### 2.2.2. Sleep Measures

##### Objective Sleep

Wrist-activity monitors (Readiband V5; Fatigue Science, Inc., Vancouver, BC, Canada) were used as an objective measure of esports athletes’ sleep. The Readiband uses a propriety algorithm to generate sleep data derived from raw acceleration signals. The Readiband has been validated against the gold standard in sleep monitoring, polysomnography, and compared against other similar consumer sleep devices [22]. This is a US Food and Drug Administration approved device for measures of physical activity and sleep [23] and has been used in performance-related sleep research, such as in the military [24], in the aviation industry [25], and with elite athletes (e.g., [26,27]). Generated sleep data included sleep onset latency (SOL), time of sleep onset, number of awakenings, wake after sleep onset (WASO), sleep efficiency, wake-up time, total sleep time (TST), and time in bed. Further, accelerometer data were used to obtain a proxy measure of circadian timing (i.e., sleep onset and sleep offset [28]) and chronotype, in lieu of self-reports (i.e., Munich Chronotype Questionnaire) or other objective markers (e.g., dim light melatonin onset).

##### Insomnia Severity Index (ISI)

The ISI [29] is a 7-item questionnaire that was used to assess the severity of insomnia in the esports athletes. Items (e.g., “How satisfied/dissatisfied are you with your current sleep pattern”) are rated on a 5-point Likert scale ranging from 0 (“very satisfied”) to 4 (“very dissatisfied”). A total score (ranging from 0 to 28) is calculated by summing individual item scores, with higher scores indicating greater insomnia severity. Morin et al. [29] demonstrated that the ISI has excellent internal consistency (α = 0.90) and sound convergent validity. A cut-off score of 10 was shown to have 86.1% sensitivity and 87.7% specificity for detecting insomnia cases in community samples [29].

##### Pediatric Daytime Sleepiness Scale (PDSS)

The PDSS [30] is an 8-item measure of daytime sleepiness. The PDSS was chosen over other popular sleepiness scales (e.g., the Epworth Sleepiness Scale [28]) due to its items showing greater face validity (i.e., questions aimed at morning sleepiness vs. situations in the ESS). The wording of items 1 and 2 were altered to increase relevance for esports athletes, as the original items were school related and no participants were attending school. Items (“How often do you fall back to sleep after being woken in the morning?”) are rated on a 5-point Likert scale from 0 (“never”) to 4 (“always”). Item 3 is reverse scored (“Are you usually alert during the day?”). Total scores range from 0 to 32 by summing item scores, with higher scores indicating greater daytime sleepiness. The PDSS has good internal consistency (α = 0.80) and is correlated with reduced sleep duration [30]. The PDSS has been used in young adults (i.e., participants up to age 24 years) [31]. A cut-off score of 15 points has been proposed to identify excessive daytime sleepiness [32].

#### 2.2.3. Mood Measures

##### Centre for Epidemiological Studies-Depression (CES-D)

The CES-D [33] is a 20-item self-report instrument used to assess symptoms of depression. Items (e.g., “I felt depressed”) are rated on a 4-point Likert scale from 0 (“rarely or none of the time”) to 3 (“most or all of the time”). Items 4, 8, 12, and 16 are reverse scored. A total score (ranging from 0 to 60) is calculated by summing individual item scores, with higher scores indicating greater levels of depressive symptoms. A score of ≥16 is used as an indicator of depression. The CES-D has excellent internal consistency (α = 0.90) and sound construct validity [34].

##### State-Trait Anxiety Inventory (STAI-Y)

The STAI-Y [35] is a 20-item measure of state anxiety. Items (e.g., “I feel calm”) are rated on a 4-point Likert scale from 1 (“not at all”) to 4 (“most of the time”). Items 1, 2, 5, 8, 10, 11, 15, 16, 19, and 20 are reverse scored. A total score (ranging from 20 to 80) is calculated by summing individual item scores, with higher scores indicating greater levels of state anxiety. The STAI-Y has good internal consistency (α = 0.86–0.95), test-re-test reliability [35], and adequate construct and convergent validity [36].

### 2.3. Procedure

The study protocol took place when all teams were actively practicing and competing in their regular seasons. The Australian and South Korean teams completed 14 days of data collection while the US team completed 7 days due to time constraints with a major upcoming tournament. Participants wore the wrist-activity monitor continuously on their non-dominant wrist for the entire observation period, except when practically unable (e.g., showering). They were instructed to maintain typical sleep–wake behavior during this time. Participants also completed a battery of self-report questionnaires on the last day of the observation period. The data were collected between 29 July and 2 November 2019.

### 2.4. Data Analysis

Data were cleaned by checking for completeness and range of values. Descriptive statistics including frequencies, means, and standard deviations were used to summarize variables. Differences among the group were analyzed using Kruskal–Wallis H test followed by Dunn’s test. Relationships between study variables (e.g., total sleep time and depression) were tested using Spearman’s rank correlation coefficients for continuous data. All study data were analyzed using SPSS 25 and *p*-values < 0.05 were considered statistically significant.

## 3. Results

Eighteen participants initially commenced the study. However, one participant from Australia was excluded because they left the team part way through the study. Thus, a total of 17 participants were included in the final analysis.

### 3.1. Demographic and Anthropometric Information

The demographic and self-reported anthropometric characteristics of participants by group are presented in Table 1. All participants were male, with the mean age being 20 ± 3.5 years (age range = 15–27 years). There were no differences in age, height, weight, and body mass index (BMI) between groups.

### 3.2. Characteristics of Esports Athletes

The participants’ esports backgrounds are presented in Table 1. All participants played First Person Shooter games for their profession; the Korean team played Overwatch, the Australian team played Counter Strike: Global Offensive, and the US team played Paladins. Participants’ career as a professional esports athlete ranged from 1.5 to 5 years.

Korean participants, on average, trained longer than US participants by 7.3 h and Australian participants by 8.6 h, totaling 13.4 h per day. The Korean team trained, on average, from 1:07 p.m. to 4:45 a.m. In comparison, the Australian team trained, on average, from 5:30 p.m. to 10:15 p.m., whilst the US team generally started their training earlier, starting at 11:48 a.m. and finishing at 6:12 p.m.

### 3.3. Sleep Characteristics

Table 2 shows the median and interquartile range of objective sleep measurements obtained from participants. Participants’ median TST was 408.4 min (i.e., 6.8 h) per night while sleep efficiency was 86.4%. Participants had prolonged WASO of 47.9 min, which was longer than the recommended 30 min [37]. Korean participants showed a median sleep efficiency of 87.5%, followed by Australian (86.3%) and US participants (86.2%) (see Table 2). There was no significant difference between groups for SOL, number of awakenings, WASO, or time in bed.

All participants had significantly delayed sleep patterns, but US participants slept earlier when compared to the other groups. Using the Kruskal–Wallis method for nonparametric test, participants from the US fell asleep significantly earlier and woke up earlier compared to the other two groups.

Over half (52.9%; *N* = 9) of the participants reported experiencing sleep disturbance such as early morning awakenings or difficulty initiating sleep before professional competitions, and four participants reported they had attempted to improve their sleep by seeking professional help (see Table 1).

### 3.4. Self-Report Measures

Descriptive statistics for self-report questionnaires (STAI, ISI, CES-D, and PDSS) are summarized in Table 3. Notably, 47.1% (*N* = 8) of all participants exceeded the clinical cut-off for insomnia (ISI scores > 10). In addition, 41.2% (*N* = 7) of participants met criteria for excessive daytime sleepiness (PDSS scores > 15).

All Korean participants exceeded the clinical cut-off of for depression (CES-D scores >16) with a significantly higher median score compared to the other groups (*p* = 0.006). Anxiety (STAI), insomnia severity (ISI), and daytime sleepiness (PDSS) did not differ between groups (see Table 3).

### 3.5. Associations between Measures

Because the data were not normally distributed, we used Spearman’s rho to calculate associations between variables (see Table 4). There was a strong correlation between CES-D and training time (hours) of the players (ρ = 0.656, *p* < 0.01). Similarly, we found that CES-D scores were strongly correlated with number of awakenings, WASO, time in bed, and wake-up time. There were no significant correlations between CES-D scores and TST.

## 4. Discussion

**Figure 1:**
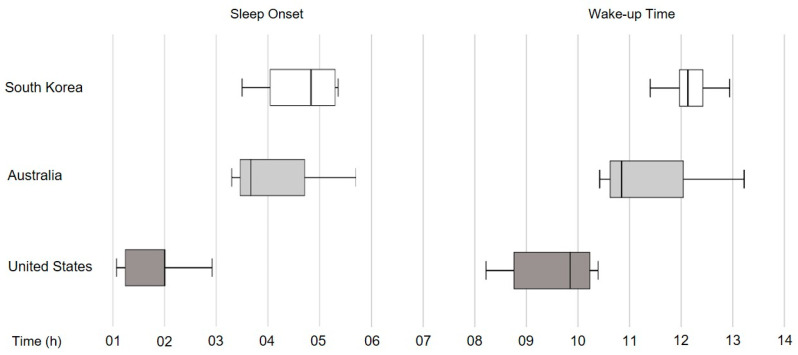
Differences in sleep onset and wake-up times.

The present study’s aims were to investigate sleep behavior and the related effect of sleep in an untapped group—esports athletes. Unlike two previous studies in this area (in the US and Germany [18,19]), our study found that participants in all three countries (US, South Korea, and Australia) had a median TST < 7 h, with prolonged WASO. Although the median sleep latency was under the clinical cut-off of 30 min [37], approximately half of the participants exceeded the clinical cut-off for insomnia, and had excessive daytime sleepiness. A striking finding was that esports athletes reported significantly delayed sleep timing—with a median sleep onset time of 3:43 a.m. and wake time of 11:24 a.m. (see Figure 1).

### 4.1. Contributing Factors to Esports Players’ Sleep

Many factors may contribute to a young person’s delayed sleep onset. In our study, one of the contributing factors appeared to be that esports athletes’ training schedules displaced their sleep opportunity. The Korean players provided the best evidence for this, with their longer training hours ranging from early afternoon to the middle of the night. It is clear that the end time of training schedules may strongly influence how late these athletes go to bed. Previous research has noted that the highest risk factors to sleep problems for esports players may be delayed sleep schedules, prolonged exposure to blue light (especially just before bedtime), and stress from competition [1,15]. There is no direct comparison group in this study, but if we consider the results of a prior study that found exposure to blue light before sleep delayed circadian timing [38], we cannot rule out the possibility that the long training time of Korean esports athletes contributed to the delay in sleep onset. Conversely, training times for US participants were much earlier, ranging from lunch time to the early evening. Hence, their sleep opportunity was not displaced to the same degree as participants from the other countries (although their circadian timing was still delayed).

Once asleep, the esports athletes experienced significant wakefulness during the night (median = 47.9 min). Given that the median ISI score for the whole sample was equal to the clinical cut-off, the prolonged wakefulness could be explained by insomnia symptoms. Preliminary evidence suggests that esports athletes (particularly First Person Shooter competitors, which comprised our sample) can experience high stress levels [39,40], which is a common precipitant for insomnia symptoms [41], However, further research using more thorough diagnostic procedures (e.g., structured clinical interview) is necessary to determine insomnia prevalence rates in esports populations.

Alternatively, as the average BMI was in the overweight range for South Korean participants, and approaching the overweight range for Australian and US participants, and daytime sleepiness was also high within our sample, we speculate the possibility of sleep-disordered breathing disrupting sleep. The nature of sedentary activity associated with esports may put esports athletes at higher risk for sleep disorders and merits further investigation.

The combination of delayed bedtimes and prolonged WASO resulted in most esports athletes obtaining less than 7 h of sleep. This is consistent with the literature in traditional sports, where many athletes do not achieve the 7–9 h of sleep per night recommended for adults [42], have poor sleep quality [43,44], and are prone to sleep disturbances around competitions [45,46]. For instance, one study reported an average sleep duration of 6.6 h in collegiate basketball players during a competitive season [47] and Sargent et al. [42] reported an average sleep duration of just 6.2 h in Olympic swimmers during a period of intensive training. Considering the well-known link between sleep and cognitive performance, the short TST esports athletes appear to experience may have performance implications for individuals whose sleep need is consistently restricted. However, it should be noted that not all individuals (adults or teens) need the recommended sleep duration for their age group [48], and therefore the performance of natural short sleepers may be differentially impacted as a result. Overall, further empirical investigation examining this link in esports is deemed critical in light of this new evidence.

### 4.2. Mood and Sleep in Esports Athletes

South Korean participants showed significantly higher depression scores than the other two groups, and importantly, all the Korean players exceeded the clinical depression cut-off score of ≥16. The CES-D score correlates well with number of awakenings, WASO, and daily training time. While it is difficult to ascertain the causality between sleep and mood from this study, there is significant literature that supports the impact of sleep on depression [49]. Additional studies with larger sample sizes will be needed to disentangle the relationship between these variables. Regardless, the present study highlights the importance of considering both sleep and mood for intervention in this population.

When we note the high correlation between depression and training time, it is conceptually possible to suggest that longer duration of training time increased depression. It is well known that working excessively long hours is significantly associated with the development of depressive symptoms [50] and intensive training is reported to cause sleep disturbances and mood changes in traditional athletes [51]. We may suggest depression is closely related to the esports athlete’s stress level. Another possibility to consider is that a large amount of training degrades the quality of sleep itself, resulting in increased depression [52]. Sleep disturbances are reported as one of the symptoms of overtraining [53,54]. During periods where training loads are high, some traditional athletes report difficulties falling asleep, restlessness during sleep, and heavy feeling in upper legs during sleep [55,56].

### 4.3. Cross-Cultural Issues Associated with Sleep in Esports Athletes

Despite the low sample size, the significant differences found between esports athletes in South Korea, Australia, and the US align with previous study findings. Young people from Asian countries (especially South Korea) obtain less sleep and demonstrate delayed sleep timing compared to their peers in Western cultures [21,57,58], as was found in the present study. Worth noting is that compared to normative data [57] the sleep onset of esports athletes was considerably later (e.g., midsleep ~05:15 vs. ~08:30; [59]). Whilst young people in the US usually obtain less sleep than their Australian counterparts [57], this was not the case here with esports athletes. The reduced sleep duration observed in North American young people is usually due to early school start times [60]. As these esports athletes were not school attenders, their sleep was not truncated. Furthermore, chronotype delays until ~20 years of age [61], and then begins to advance in its timing. As the esports athletes from the US in our sample were slightly older (~22 years) than their Australian and South Korean peers (~19 years) this may be one factor contributing to their earlier sleep mid-point.

### 4.4. Chronotype and Performance

The strong preference for an evening-type (i.e., late chronotype) sleep pattern found in our study has potential implications for esports athletes’ sleep and performance. Research into the effect of chronotype on athletic performance has recently increased [62]. Although some studies have observed no effect [63], other studies have shown a relationship [64,65]. For example, in one study evaluating chronotype and training time in a group of swimmers, it was reported that morning-types and those who habitually train in the morning were faster, less fatigued and more energetic during morning training sessions. Conversely, neither-types and those who regularly train in the evening felt more energetic and less fatigued during evening training sessions [65]. Hence, it would appear that an individual’s chronotype and training schedule could affect performance, and it may be important to consider individual chronotypes depending on competition timing. In terms of esports, evening-type esports athletes who have evening training and matches are less likely to experience sleep and performance impairments, as their competition and chronotype are aligned. However, for earlier-scheduled matches, there would be a greater risk for both acute sleep loss (due to being unable to sleep in to a typical normal wake-up time) and possible performance decrements associated with being awake and needing to perform close to the core body temperature nadir [66,67].

### 4.5. Clinical Implications

Sleep interventions designed to enhance sleep and performance outcomes of esports athletes appear warranted, especially given the low number (23.5%) of players who previously attempted to improve their sleep but failed. Sleep education (both verbal and written) should be tailored to esports players to enhance relevance and promote active engagement [5,68]. Given the risk for poor sleep hygiene (e.g., proclivity for late night technology use and regular caffeine use), sleep education should include sleep hygiene recommendations, which pertinently for esports athletes, has been found to be useful for traditional athletes following late-night matches [69]. The typical apathy toward sleep-related behavioral change observed in young people might indicate the inclusion of motivational strategies to enhance sleep treatment compliance [70].

The delayed sleep timing of participants found across all countries highlights the importance of incorporating chronobiological strategies, including the use of blue-light-blocking glasses following late training sessions [71], exogenous melatonin administration at the beginning of the sleep period, and scheduled light exposure at the end of it [72], as well as team-based activities (e.g., eating breakfast together) that anchor sleep/wake behavior [73]. Other team social cues could also be implemented by team coaches, such as set training times, which would help regulate sleep timing. Cognitive and behavioral techniques could prove useful [15]—for example, mindfulness-based stress reduction has been helpful for younger people’s sleep [74,75].

### 4.6. Limitations

The current study had a ‘small’ sample with 17 participants. Yet, it should be noted that elite athletes of any kind are very difficult to recruit, which is why in traditional sports science research, small sample sizes are common (e.g., [76]) and considered acceptable, including in landmark studies such as Mah et al. [47]. Moreover, the two previous studies [18,19] that measured sleep of esports athletes had smaller samples than the present study. However, further studies will be needed to complement our findings in order to enhance generalizability. An additional sampling consideration is that although available normative data offers a useful comparison, the inclusion of a control group would enable a more direct comparison between esports athletes and non-esports athletes. For this study, actigraphy was used to analyze objective sleep data. Considering previous studies that show significant discrepancies may exist for WASO and TST between objective and subjective sleep data [77], further studies using both actigraphy and sleep diary data may be necessary (including monitoring daily alcohol and medication use). Furthermore, some criticism has been made regarding the confidential nature of the mathematical modelling that underpins the propriety algorithms used in wrist activity monitors [78] such as Readibands, Polar and Garmin. However, we note that ‘big data’ and smaller scale studies that use wrist-activity monitors with automated propriety algorithms are increasingly being published in reputable journals within the sleep literature (e.g., [21,79]). Finally, because we did not include performance measures, we were unable to determine how these sleep patterns influence performance in esports.

## 5. Conclusions

Findings from the present study indicate that esports athletes have delayed sleep patterns, experience prolonged wake after sleep onset and obtain <7 h sleep per night. These sleep patterns may be associated with mood (depression) and training time in some esports athletes, particularly those from South Korea. Future research should aim to measure the impact of sleep on performance in esports, and design sleep interventions specifically tailored for esports athletes and the conditions that characterize competing at elite levels in esports.

## Figures and Tables

**Table 1:** Characteristics of the study participants.

Group	Total (*n* = 17)	South Korea (*n* = 8)	Australia (*n* = 4)	United States (*n* = 5)	*p*
**Measure**					
Age ^a^	20.0 ± 3.5	19.0 ± 3.3	19.4 ± 2.9	22.1 ± 3.8	0.284
% of male ^b^	100.0%	100.0%	100.0%	100.0%	
BMI ^a^	24.7 ± 16.8	25.2 ± 16.8	24.6 ± 15.8	23.9 ± 23.3	0.742
Years as a professional esports athlete ^a^	2.44 ± 1.32	1.74 ± 1.06	2.50 ± 1.00	3.50 ± 1.41	0.052
Training Hours per day ^a^	9.21 ± 4.36	13.38 ± 2.00	4.75 ± 0.96	6.10 ± 1.34	0.002 **
Sleep disturbance before competition ^b^	9 (52.9%)	4 (50.0%)	3 (75.0%)	2 (40.0%)	0.564
Attempts to improve sleep (*n*) ^b^	4 (23.5%)	2 (25.0%)	1 (25.0%)	1 (20.0%)	0.976
Caffeine dose (mg) ^a,c^	114.7 ± 118.3	150 ± 162.6	100 ± 57.7	70 ± 44.7	0.648
Sleep medication use (*n*)	0 (0.0%)	0 (0.0%)	0 (0.0%)	0 (0.0%)	

^a^ Data are expressed as mean ± standard deviation and analyzed by Kruskal–Wallis test. ^b^ Data are expressed as number of analysis of χ^2^-test for categorical variables, ^c^ the median of each multiple-choice presented response was used to calculate the caffeine dose, ** indicates correlations significant at *p* < 0.01 levels (2-tailed).

**Table 2:** Difference between groups in objective sleep data.

Measure	Group				*p*-Value
	Total	South Korea	Australia	United States	
TST (min)	408.4 (386.4–444.1)	410.4 (400.1–444.6)	413.6 (382.3–422.9)	404.3 (345.0–454.2)	0.975
SOL (min)	20.4 (13.5–31.9)	16.3 (12.5–35.7)	21.4 (13.4–27.6)	26.6 (15.9–46.3)	0.573
NWAK (*n*)	4.4 (3.4–6.4)	5.1 (3.3–6.8)	3.6 (3.2–5.8)	5.1 (2.9–7.8)	0.663
WASO (min)	47.9 (27.6–65.3)	50.5 (26.9–67.2)	31.8 (26.2–68.7)	55.0 (30.0–90.0)	0.770
TIB (min)	505.5 (480.4–542.1)	513.2 (476.9–552.2)	493.9 (477.9–509.4)	510.0 (480.0–553.8)	0.594
SE (%)	86.4 (86.3–87.5)	87.5 (86.7–87.9)	86.3 (86.1–86.8)	86.2 (86.0–86.7)	0.019 *
SO (hh:mm)	03:43 (02:28–05:06)	04:50 (03:58–05:18)	03:40 (03:23–05:06)	02:00 (01:09–02:28)	0.005 **
WT (hh:mm)	11:24 (10:19–12:14)	12:08 (11:56–12:29)	10:51 (10:31–12:38)	09:51 (08:29–10:19)	0.004 **

Data are expressed as median and interquartile range, and analyzed by Kruskal–Wallis test. * Indicates correlations significant at *p* < 0.05 levels (2-tailed) and ** indicates correlations significant at *p* < 0.01 levels (2-tailed). Abbreviations: TST, total sleep time; SOL, sleep onset latency; NWAK, number of awakenings; WASO, wake after sleep onset; TIB, time in bed; SE, sleep efficiency; SO, sleep onset; and WT, wake-up time.

**Table 3:** Difference between groups in psychological measures.

Group	Group				*p*-Value	Post-Hoc
	Total	1. South Korea	2. Australia	3. United States		
CES-D	22.0 (12.5–27.5)	27.5 (24.5–34.8)	10.5 (6.5–19.0)	13.0 (11.0–23.5)	0.006 **	1 > 2, 3
STAI	39.0 (35.5–42.5)	39.0 (34.5–40.0)	37.5 (30.3–44.8)	39.0 (36.5–52.0)	0.443	
ISI	10.0 (6.0–15.0)	10.5 (7.3–15.5)	10.5 (3.0–14.3)	9.0 (3.5–16.5)	0.866	
PDSS	15.0 (10.5–17.5)	16.5 (11.3–21.0)	14.5 (9.3–17.5)	13.0 (10.5–15.0)	0.109	

Data are expressed as median and interquartile range, and analyzed by Kruskal–Wallis test. ** Indicates correlations significant at *p* < 0.01 levels (2-tailed). Abbreviations: CES-D, Centre for Epidemiological Studies-Depression; STAI, State-Trait Anxiety Inventory; ISI, Insomnia Severity Index; PDSS, Pediatric Daytime Sleepiness Scale.

**Table 4:** Non-parametric correlations between variables measured.

	1	2	3	4	5	6	7	8	9
1. ISI									
2. CES-D	0.58 *								
3. STAI	0.48	0.32							
4. PDSS	0.54 *	0.67 **	0.15						
5. SOL	−0.07	0.02	0.19	−0.26					
6. WASO	0.79 **	0.52 *	0.46	0.42	0.12				
7. TST	−0.21	0.01	−0.12	0.17	−0.20	−0.30			
8. SO	−0.10	0.27	−0.14	0.43	−0.24	−0.26	−0.01		
9. WT	0.21	0.47 *	−0.04	0.68 **	−0.39	−0.01	0.23	0.85 **	
10. Training time	0.16	0.66 **	0.09	0.29	−0.23	−0.02	−0.15	0.50 *	0.46

The correlation is presented as *r_s_.* * Indicates correlations significant at *p* < 0.05 levels (2-tailed) and ** indicates correlations significant at *p* < 0.01 levels (2-tailed). Abbreviations: CES-D, Centre for Epidemiological Studies-Depression; STAI, State-Trait Anxiety Inventory; PDSS, Pediatric Daytime Sleepiness Scale. SOL, sleep onset latency; WASO, wake after sleep onset; TST, total sleep time; SO, sleep onset; and WT, wake-up time.

## Data Availability

The data presented in this study are available on request from the corresponding author. The data are not publicly available due to participants’ privacy protection.
